# Austerity measures and the transforming role of A&E professionals in a weakening welfare system

**DOI:** 10.1371/journal.pone.0212314

**Published:** 2019-02-13

**Authors:** Angeliki Kerasidou, Patricia Kingori

**Affiliations:** The Ethox Centre and The Wellcome Trust Centre for Ethics and Humanities, Nuffield Department of Population Health, University of Oxford, Oxford, United Kingdom; University of Kent, UNITED KINGDOM

## Abstract

In 2010, the UK embarked on a self-imposed programme of contractionary measures signalling the beginning of a so-called “age of austerity” for the country. It was argued that budgetary cuts were the most appropriate means of eliminating deficits and decreasing national debt as percentage of General Domestic Product (GDP). Although the budget for the National Health Service (NHS) was not reduced, a below-the-average increase in funding, and cuts in other areas of public spending, particularly in social care and welfare spending, impacted significantly on the NHS. One of the areas where the impact of austerity was most dramatically felt was in Accidents and Emergency Departments (A&E). A number of economic and statistical reports and quantitative studies have explored and documented the effects of austerity in healthcare in the UK, but there is a paucity of research looking at the effects of austerity from the standpoint of the healthcare professionals. In this paper, we report findings from a qualitative study with healthcare professionals working in A&E departments in England. The study findings are presented thematically in three sections. The main theme that runs through all three sections is the perceptions of austerity as shaping the functioning of A&E departments, of healthcare professions and of professionals themselves. The first section discusses the rising demand for services and resources, and the changed demographic of A&E patients—altering the meaning of A&E from ‘Accidents and Emergencies’ to the Department for ‘Anything and Everything’. The second section in this study’s findings, explores how austerity policies are perceived to affect the character of healthcare in A&E. It discusses how an increased focus on the procedures, time-keeping and the operationalisation of healthcare is considered to detract from values such as empathy in interactions with patients. In the third section, the effects of austerity on the morale and motivations of healthcare professionals themselves are presented. Here, the concepts of moral distress and burnout are used in the analysis of the experiences and feelings of being devalued. From these accounts and insights, we analyse austerity as a catalyst or mechanism for a significant shift in the practice and function of the NHS–in particular, a shift in what is counted, measured and valued at departmental, professional and personal levels in A&E.

## Background

Since 2010, many European countries have introduced austerity measures. These were ostensibly to address budget deficits caused by the 2008 stock market crash and subsequent nationalisation of private debt. Austerity was presented by many international bodies and local governments as the only option [1–3]. Yet a number of economists have argued that austerity policies hindered rather than aided recovery [4–7], and international institutions, such as the UN, have described austerity more as a political choice rather than an economic necessity [8, 9]. While this debate is ongoing, economic analyses and statistics illustrate the effects reductions in public spending have had in many European societies, including the UK [10–14]. These retrospective and prospective economic and statistical analyses are important in projecting and demonstrating the consequences of financial cuts. However, they cannot capture the full picture of austerity, as they often lack insight into the everyday meaning and experience of austerity for a range of actors.

In this paper, we seek to focus on the perspectives of healthcare professionals tasked with providing public services, with an emphasis on how budgetary constraints shape their sense of purpose, daily decision-making and practice. The healthcare professionals under examination are those employed in Accident and Emergency (A&E) departments in a variety of locations in England. We demonstrate that while statistics are valuable in capturing macro-level trends, they need to be accompanied by detailed accounts from micro-level actors in public sector settings to gain a more comprehensive understanding of what austerity means in practice.

### Austerity and its measured impact on the social ecosystem and healthcare in the UK

In 2010, the UK embarked on a self-imposed programme of contractionary measures signalling the beginning of a so-called “age of austerity” for the country [15]. It was argued that budgetary cuts were the most appropriate means of eliminating deficits and decreasing national debt as percentage of General Domestic Product (GDP) [16]. Austerity measures have been imposed on all areas of UK public spending, including welfare and social security, education and local government funding. The original stated plan was to end austerity by 2015–2016. However, in 2017, after a snap general election, the government confirmed its decision to extend austerity policies indefinitely [17]. More recent developments suggest a move towards ending austerity as an official programme [18, 19], but whether this will be possible still remains uncertain [20].

A number of studies and reports illustrating the effects of austerity policies of the past eight years reflect a disproportionate impact on lower socio-economic groups [11, 21, 22], with cascading effects on social services and the ecosystem of service provision and support available for the poorest in the UK [9, 10].While the relationship between poor socio-economic conditions and poor health outcomes remain relatively uncontroversial [23, 24], correlations between austerity, budgetary cuts and the UK’s healthcare system remains inconclusive and speculative at the level of government. For instance, the UK Government has denied that austerity is correlated with challenges in the NHS to meet increasing demands on its services with ever-shrinking resources. In an address to the House of Commons in February 2016, the then Health Secretary, Jeremy Hunt, suggested that concerns about the NHS were overstated, and defended austerity policies by saying that: *“I do think it’s stretching it a bit to call this an austerity driven problem when we’re putting next year the sixth biggest increase in funding for the NHS in its entire 70-year history”* [25].

The Government’s focus on the amount invested rather than percentage increase stands in contrast to the many academic studies and reports of this issue which have demonstrated the negative effects of austerity on the NHS [26, 27]. The main conclusion of these studies is that although, in 2010, the UK Government promised to protect the NHS budget, this promise was not met in real terms [28]. Between 2010 and 2014, the NHS budget was increased only by 1.3% as opposed to a 4% increase in previous years [10], despite the notable rise in demand spurred to a large extend by budgetary cuts elsewhere. For example, funding for mental health services decreased by 8% in real terms between 2010 and 2015 and 2,100 beds were lost due to the closure of NHS mental health facilities during the same period [29]. Also, as the British Medical Association (BMA) notes, funding cuts to local governments and the decrease in the available budget for social care have had a significant impact on the health and wellbeing of citizens and their access to services [30]. Between 2010 and 2015, it is reported that local Councils in England lost approximately 27% of their budget, which had serious effects on their ability to provide necessary care to their residents [31].

After eight years of record-low budget increase for the NHS, in 2018, the Government announced an increase in the NHS budget by £1.6bn and an extra £540million from the Department of Health and Social Care. This signifies a 2.4% increase in the NHS budget, a growth from that of previous years, but still short of the 4% historic average [32]. The budget for primary care is also set to rise by £2.4bn by 2020/21, with plans to recruit 5000 more General Practice (GP) doctors by the same time, and offer universal access to GP appointments at evening and weekends to all by March 2019 [33]. The Government also repeated its commitment to improve mental health care in the UK by asking Clinical Commissioning Groups (CCG) to increase their spending on mental health [32], and by requiring better integration between mental health and primary care [33]. What the NHS Forward Plan and new Mandate omits are plans to address issues regarding recruitment, retentions and workforce capacity [32]. The quarterly report by NHS Improvement published in September 2018 highlighted increasing medical vacancies as a major concern for the safe and efficient operation of NHS facilities, citing increased demand and high leaver rates [34].

### Austerity and its measured impact on A&E services

In A&E departments patient attendance has risen substantially. From 2011, the overall number of patients attending A&E grew by 1.9 million. A greater increase was observed in A&E walk-in centres and minor-injury units when compared to major and specialist A&E units. By 2013, three years after the UK government’s austerity measures began, the majority of A&E departments in the UK were reporting significant increases in admissions. This meant that many departments were missing their four-hour target, a measure of efficient patient care. The four-hour target, first introduced in 2000 by the Blair government, indicates the absolute maximum time deemed acceptable to keep emergency patients waiting before being seen by a healthcare professional [35]. Meeting targets, particularly the four-hour target, is an imperative for all A&E departments, as their performance and subsequent funding is heavily dependent on their compliance.

The NHS responded to the A&E crisis by commissioning an independent review, and in November 2013 the Keogh Report was published [36]. The Report found that up to 40% of A&E admissions where discharged without treatment, which suggested that these patients were using A&E for services, which might not be deemed an emergency [37]. It is worth noting here that a subsequent study that tried verify this claim found that 85% of the patients presenting in A&E were there appropriately, and the majority of the other 15% were cases of young children [38]. However, based on the Keogh Report, the NHS introduced a campaign to deter non-emergency patients from attending A&E. The campaign’s goal was to educate the public about the appropriate use of healthcare facilities and to re-direct patients with urgent but not life-threatening conditions away from A&E, so that it could focus on ‘*genuine* life-threatening emergencies such as … *severe* chest pain, *severe* bleeding, *severe* burns …’ [39]. Yet, despite the changes in the provision of emergency care launched after the Keogh Report, A&E departments around the UK continued performing poorly against stated targets [40]. Also, in 2015, a price-cap was introduced on agency healthcare workers to reduce NHS costs, which drastically curtailed departments’ ability to hire additional staff [41]. NHS Improvement reported that thousands of breaches of the price-cap took place between July and September 2016, as hospitals tried to secure the staff necessary to keep their departments functioning [42]. At the same time, below-inflation salary increases since 2010 for non-agency staff meant a significant decrease in their earnings [43].

### Austerity and healthcare: Measuring the everyday experiences of healthcare professionals

Few studies explore the impact of austerity policies in healthcare from the perspective of healthcare professionals, and the majority focus on other European countries. In Spain, a qualitative study among healthcare professionals reported that austerity had a negative effect on the operation of the healthcare system, the quality of service provision and on the ability of patients to access services [44]. In Belgium and Netherlands, a study looked at the effects of austerity policies on the provision of elderly care, concluding that lack of resources have had a negative impact on the care of this population [45]. Even fewer are the studies that have focused on the effects of austerity measures on healthcare professionals themselves. A small number of studies examined how healthcare professionals chose to migrate [46, 47] or shift from public to private sector employment in their countries as a response to the financial crisis on their personal and professional lives [48]. A qualitative study examining the experiences of healthcare professionals in Greece found that despite the significant impact austerity-driven policies and reforms on these professionals, they displayed resilience and desire to adhere to what they perceived to be their moral and professional duties towards their patients [49].

In the UK, considerable media attention has been given to the impact of austerity measures on the NHS. In addition, institutions such as the King’s Fund and the Nuffield Trust have been conducting studies and publishing reports on this topic [28, 50–53]. A systematic review examining psychiatric morbidity among doctors in the UK found that ‘*a government-driven emphasis in the NHS on performance management and targets …[and] the current climate of austerity in the UK*’ has further increased the exposure of doctors to stress and burnout leading to increased psychiatric morbidity amongst these healthcare professionals [54]. However, these findings were not obtained from in-depth qualitative examinations with healthcare professionals. To our knowledge, there are no published studies which have focused attention on the effects of austerity on the everyday experiences of healthcare professionals in the UK, on how austerity policies are shaping the provision of healthcare, as well as the professional character of the medical and nursing professions. This paper seeks to attend to this gap in the literature.

## The study

This study intended to investigate the experiences of austerity cuts in healthcare, and to move beyond the aforementioned numbers and statistics to capture and analyse the perspective of frontline healthcare professionals. It focused on A&E, which is regarded as the ‘front-face’ and ‘barometer’ of the NHS and as such, it is considered as an important indicator of how the rest of the NHS is functioning [55]. Qualitative interviews were conducted with doctors and nurses working in A&E departments in hospitals across England. Semi-structured interviews were employed to capture their personal insights and experiences in the provision of healthcare under austerity conditions. Paying attention to the views of frontline healthcare professionals in A&E not only allowed for their valuable experiences to be considered but also potentially provides insights into some important themes of possible relevance to other NHS departments.

## Methods

Between February and November 2017, sixteen semi-structured interviews were conducted in three A&E departments in Central, South-East and North-West England. These A&E departments represented a diverse range of sizes and locations (e.g. different parts of England and different urban and rural populations). They also represented different types of hospitals with two of the A&E departments in this study being based in teaching hospitals and one in a regional hospital. The interviews involved nine doctors and seven nurses of difference seniority and years of experience in working for the NHS.

Participants were recruited via email, which was forwarded to the departmental mailing list by the local Research Nurse or Clinical Lead. Posters were also distributed around A&E departments inviting staff to contact the research team directly if they wished to participate. In addition, on two occasions, the researchers were invited to Departmental meetings by the Research Nurse, or Clinical Lead, who acted as the main hospital contact and gatekeepers. Staff interested in participating would then email the researchers and arrange a convenient date and time for the interview to take place.

Fourteen face-to-face interviews were conducted, in private rooms in the various hospitals, and two were conducted via Skype. The place and time of interview was arranged to accommodate the schedule of participants and this is reflected in the time, location and length of the interviews. The majority of interviews with junior doctors and nurses were conducted during lunch breaks and after their shifts. Some members of staff conducted interviews on their days off. For senior members of staff, such as consultants and senior nurses, interviews were conducted during their working day and were often arranged as meetings in their schedules.

The concept of austerity was mentioned in the Participant Information Sheet (PIS) circulated to prospective participants but no formal definition of austerity was offered by the researchers. At the time of this study, the UK was already in the sixth year of austerity, and the concept was widely used and discussed in the public and political discourses. Yet, as mentioned earlier in this paper, whether austerity measures have been imposed and what constituted austerity in the NHS are controversial issues. For this reason the researchers decided not to offer a definition of austerity to allow participants to confirm, deny, challenge and offer their own reflections on the topic. During interview, participants were asked to describe and reflect on their everyday experiences of providing care in A&E particularly since 2010–2011 and to provide their own accounts of any changes in experience identified. Participants who had been employed by the NHS for many years were able to reflect on the differences before and after 2010. This approach aimed at allowing participants to highlight and reflect on areas of importance regarding their working conditions, sense of purpose and motivations in the conduct of their roles.

Interviews lasted approximately 45 minutes, ranging between 33 minutes and 80 minutes in length. All interviews were recorded, transcribed verbatim, anonymised and analysed using thematic analysis. Each researcher (AK and PK) read the transcripts before coding them separately. Sections of coded transcripts were then discussed to identify areas of similarity and differences in coding. This process formed an important feature of data analysis and interpretation. The final themes were agreed upon by both researchers.

### Ethical clearance

This project received ethical and scientific approval from: Central University Research Ethics Committee (CUREC; R43862/RE001), Clinical Trial and Research Governance (CTRG), Health Research Authority (HRA; IRAS 213721), and from the Research and Development (R&D) offices of each hospital involved, a process which began in July 2016 and was completed in November 2017. Since recruitment occurred within NHS facilities, ethical approval from CTRG, HRA and from individual R&D departments were required before research participants could be approached. These authorities are primarily tasked with reviewing and authorising clinical trials, which is reflected in the design of their forms and presents a challenge for qualitative research. The only concern raised by the CTRG was how to deal with potential revelation of wrongdoing by healthcare professionals, which would be deemed to pose a direct and immediate risk to the health and safety of patients. By December 2016 the project was approved by CUREC, CTRG and HRA. In the majority of cases the communication with R&D departments was straightforward. Access was readily obtained, and recruitment started in February 2017. However in one site, the A&E department’s Clinical Lead, after months of waiting, declined their support citing time constrains. Without the support of the Clinical Lead, the R&D office also withdrew their approval. At a second site, the R&D department was slow in communication and a number of times asked for minor amendments to the HRA and CTRG forms, a process that would take few weeks each time to complete. By November 2017, researchers were still trying to get final approval from the R&D office, by which time a decision was made to withdraw the request, as time on the project was running out.

The process of obtaining ethical clearance from these different bodies played an important role in gaining access to hospitals and also the length of data collection. Also, the use of various gatekeepers ultimately shaped the composition of research participants, as the refusal from a Clinical Lead meant that all the staff in that department could not take part in the research independent of their interest in the subject. As a consequence, there are numerous ways in which the interviews obtained were shaped by external factors, from the efficient R&D response in hospitals, which expedited the process, to the agreement or refusal of the Clinical Lead.

## Results

The study findings are presented thematically in three sections in this paper. The main theme that runs through all three sections is the perceptions of austerity as shaping the functioning of A&E departments, the healthcare professions and professionals themselves. The first section discusses the rising demand for services and resources, and the changed demographic of A&E patients—altering the meaning of A&E from ‘Accidents and Emergencies’ to the Department for ‘Anything and Everything’. The second section in this study’s findings, explores how austerity policies are perceived to affect the character of healthcare in A&E. It discusses how an increased focus on the procedures, time-keeping and the operationalisation of healthcare are considered to detract from values such as empathy in interactions with patients. In the third section, the effects of austerity on the morale and motivations of healthcare professionals themselves are presented. Here, the concepts of moral distress and burnout are used in the analysis of the experiences and feelings of being devalued. From these accounts and insights, we analyse austerity as a catalyst or mechanism for a significant shift in the practice and function of the NHS–in particular, a shift in what is counted, measured and valued at departmental, professional and personal levels in A&E.

Table 1. Below provides a summary of the themes and subthemes presented and discussed in this paper.

**Table 1:** Table showing the main themes and subthemes along with representative quotes.

**THEME**	**SUBTHEME**	**REPRESENTATIVE QUOTES**
**Effects on the A&E Departments**	What is counted	*“We see more people than we ever saw and the* ***resources haven’t increased in line with that demand***.*” (Nurse*,*1)* *“However what happens is … we become*, ***A&E stands for anything and everything***, *not accident and emergency I think*, *its anything and everything*, *just send it to A&E*. *GPs who run out of things they can do with patients*, *they send them to A&E*, *Police if there are patients acting strangely they send them to A&E; or I am feeling a bit worried about my cough*, *I have to go to A&E*. … *That is a big problem for A&Es at the minute*.*” (Nurse*,*12)*
**Effects on healthcare profession**	What is measured	“So it’s (the four-hour target) not an indicator of quality at all. […] it’s a bit of waffle isn’t it? And you could say, right ok, you are good quality because you get hardly any complaints, or incidents, or most patients have a safe journey through the A&E department. All those things are really important quality indicators. **So the performance target is just a measure of time. It is not a measure of anything else.** And they use it because it is easy to measure. **But you could argue that if people spend longer in the department, then they get more treatment, we may have more time to spend with them. They may get a better deal. So a four-hour target itself is not a quality measure.** (Doctor, 9)
**Effects on healthcare professionals**	What is valued	Every time that we go into a week knowing we are five-six doctors short because they would normally be filled by locum agencies and we can’t fill them because the Trust won’t pay money, or there isn’t the budget for supply doctors and we can’t recruit. **It’s just about feeling that actually our time, our own personal life is valuable, time with our families is valuable and we cannot expect to do over our contract every single working week, and increasingly so**. […] I think that’s really important to get across. **But yes we feel undervalued, but it’s not a monetary thing, it’s a recognition of our skill mix and a recognition of the stress which is upon us and the recognition of us as being human and having lives, which exist outside of the health service, which I think have long been forgotten by many people**.” (Doctor, 8) [Emphasis added]

The first central theme to emerge from our data describes healthcare professionals’ personal accounts of the effects of austerity on the everyday operation of A&E. Doctors and nurses provided first-hand accounts about how budgetary cuts and restrictions in health and social care in general affected the functioning of A&E departments. Many referred to A&E as the *‘front face of the NHS’* and used the analogy of *‘the canary in the mine’* to describe the interconnectedness between various areas of healthcare, and of explaining why changes elsewhere in the public health sector were having a significant impact on the functioning of A&E.

## Effects on the A&E departments: What is counted

When healthcare professionals discussed their personal experiences of working in A&E they acknowledged that emergency medicine is, by nature, a busy and hectic speciality with consistently scarce resources and high degrees of time pressure. However, since 2010 they observed their daily experiences to have worsened due to growth in demand for services without an equivalent or satisfactory increase in available resources (in particular, human, monetary and time) given as the main explanation. For instance, increase admissions and a cap on agency staff, introduced in 2015 as a way of reducing expenditures, meant that A&Es were finding it difficult to operate effectively and were struggling to cope.

In the following interview extract, Nurse 14 explains staff shortages and its effects in their department:

…we can’t get [staff] cover anymore and we are not allowed to go out to agencies [to get additional staff], because it costs too much money. So **we are at risk as nurses of working down quite a lot, like this morning six nurses down and an extremely full department**. Resus A, which is our top bay for cardiac arrest traumas, so everything was full. And I am sure if we had the viewing room, which is where we put deceased patients, I am sure that would have been full if we’d let them.” (Nurse, 14) [Emphasis added]

As this quote demonstrates, agency staff are an essential part of the service delivery. The financial cost they impose on departments is captured through financial audits and this is seen to be given more weight than the service and support that they provide. In this way, their increased absence was linked to a cascading effects of staff shortage in departments.

The limited access to additional staff and the increase in admissions were not the only reason why those working in A&E considered themselves to be challenged by austerity measures. There was also a lack of available beds in the various speciality wards, therefore patients could not be transferred to other wards and their presence in A&E meant that resources for newly admitted patients were compromised. So that, in the event that the number of beds in A&E were not reduced, A&E staff still feel the effects of being not being able to transfer patients elsewhere. This insight, from those interviewed, emphasises the importance of examining the impact of austerity holistically. Merely counting the number of beds in an A&E department does not fully capture what was happening elsewhere in the hospital and how the hospital ecosystem influences the functioning of A&E—on patient care and staff’s ability to perform their roles to what they considered to be a satisfactory standard. As noted earlier, it was not only other departments in a hospital that shaped service delivery in A&E but other areas of the welfare system too; and these effects, even though difficult to quantify, were felt by frontline healthcare staff. For instance, A&E doctors and nurses acknowledged that during the winter months, it is not uncommon for A&E to be operating at full capacity. However, this occurrence had recently become much more frequent and extended beyond the seasonal variations that they previously observed. Doctor 4 describes what they experienced to be *‘the normal now for the ED*’ which is a *‘permanent red alert’* everyday as they experience a growing number of patients that ‘*have nowhere to go*’:

So often we start in the morning in the ED [Emergency Department] **at eight o’ clock in the morning and every single bed is already full with patients** that have been referred to the medical team or the surgical team or whatever, **but have nowhere to go**. So that means in the ED that we are having nowhere to see the patients who are still coming in … **everybody queuing up in the corridors and ambulances can’t deliver anyone.** […]And that used to be the case…in the winter when it would be bad but **now it seems to be every day**. **That’s the normal now for the ED.** So it’s gone from a situation of intermittent overload and a sort of red alert situation, to being on fairly permanently, what feel **like permanent red alert**. (Doctor, 4) [Emphasis added]

In this way, inadequate funding or direct cuts in services outside the hospital environment are described as having a marked increase on the number of A&E patients which, in turns, effects service delivery. This following quote from Doctor 8, supports data reported earlier in this paper that cuts in social welfare are closely linked with increased demands in healthcare. Here they describe the effects on A&E resources from personal observation over the last ten years:

[A]ttendances in all emergency departments have increased substantially over the last 10 years due to cuts in other services, such as reduction in GP working hours, reduction in social care facilities due to budget cuts, reduction in mental health facilities, again you can trace back to budget cuts, less mental health beds, less community hospital beds, etc. So all those **healthcare related resources have suffered I think from austerity measures throughout the last 10 years and the pinnacle of that if you like is that we now have about 20%-30% more attendances in emergency departments than we did 10 years ago.** […] there is never enough staff to see the number of patients coming in through the door, therefore **it is very hard work and sometimes it’s relentless…**” (Doctor, 8) [Emphasis added]

As discussed earlier, the correlation between poor socio-economic conditions at a societal level and increased demands in healthcare services is well-supported in the academic literature. This doctor suggests that cuts in services elsewhere in the welfare system has produced increases of between 20–30% in admissions but this impact was not being systematically measured. Rather, what is measured and counted is the number of patients that were attending A&E “inappropriately”, as in the case of the Keogh Report discussed above. Yet, the impact of underfunding elsewhere in the welfare system was felt and experienced by those on the frontline.

Another indication of the major impact of funding cuts elsewhere in the system is the change in the demographic of patients presenting in A&E. All those interviewed mentioned that elderly and mentally ill patient groups regularly and increasingly turn to A&E for help, even though, they argued, this is not the appropriate place for them to receive care. In the following quote, Doctor 7 describes how their A&E department has been increasingly tasked with caring for both severe and acute emergencies but also patients that, according to their opinion, should have been treated elsewhere:

The **austerity cuts have not really affected us in the front line. What they have affected is every other part of the system.** So we are funded reasonably well. But we are funded to do not our job, **we are doing everybody else’s job now**. Social services have collapsed as far as I can see, you know, to all intents and purposes, it doesn’t work. **Elderly care is awful, it is dire in this country. Psychiatric care is dire.** Things that don’t hit the press, so the elderly care, mental health care are not funded and **the patients end up in the emergency department**. (Doctor, 7) [Emphasis added]

There are a number of interesting points raised in this quote. One of the most striking features of the quote is its seemingly contradictory nature. It begins by stating that austerity has not affected A&E and then goes on to outline the ways in which it has done so. This apparent contradiction exposes the importance attached to what is measured. If a purely financial examination is undertaken, because the funds provided to this A&E department have remained the same and it continues to be well funded, it would appear that austerity has not had an impact on its A&E services. Yet, because of what this doctor describes as the “collapse” of social services there is an increase in workload and a change in the type of conditions being presented which are harder to capture and not easily measured.

The changing role of A&E from attending to short term acute conditions to a department increasingly tasked with patients that challenge the scope and training of many frontline staff, is a recurring theme in the accounts of those interviewed. Some of the most vulnerable patients and those in need of the greatest care are presenting in greater numbers. So, it is not simply an increase in the number of patients, but also an increase in a certain type of patient with *specific conditions*, which was having an impact on everyday service delivery. Although official reports often mentioned the aging population as a contributing factor for the increase admissions in A&E [36, 56], what was not measured in these official reports was how decreasing social care services and local authorities shrinking budgets were contributing to the rise in numbers of elderly patients presenting in A&E. According to a Doctor 8, A&E is increasing being used by the elderly, because ‘they have nowhere else […] to go’:

**More and more elderly people are brought to hospital because there is nowhere else for them to go** […] There aren’t day centres, there isn’t community hospitals, there isn’t community care at home for them.” (Doctor, 8) [Emphasis added]

In addition, the following quote obtained from a nurse provides greater insight into the resources diverted into caring for the mentally ill in A&E but also that these vulnerable patients were being poorly served by not having adequate follow-up and aftercare in an environment not designed for such services:

**There are no beds and they (mental health patients) end up staying here for three-four days whilst we try to find a bed somewhere across the country. And there is also the safety thing.** Again if you are looking after eight patients and one of those is that patient, and that patient leaves the department, and you don’t see them because you are looking after another one, that patient is vulnerable and you lost them, you are responsible for them. But then how can you be expected to be responsible in an open area? It’s happened! **Patients are supposed to be sectioned and looked after here… the nurse has got distracted with other patients, they‘ve absconded, and they killed themselves.** It has happened. …. And I feel for that person who must feel awful because it was their responsibility to look after that patient. But what can you do! It’s an open area. What can you do! It’s not appropriate to look after patients who are that vulnerable in ED. But they cannot go up to the ward because they are not under medics, **they have to stay in ED, you are in Catch 22! There is nowhere else for them to go.** (Nurse, 3) [Emphasis added]

All study participants agreed that increasing the funding for A&E departments, although welcome, could not compensate for inadequate funding elsewhere in the welfare system. Rather, they argued that efforts needed to be focused on enhancing and boosting other care provision services, such as primary, social and mental health care. These quotes clearly demonstrate that the types of patients presenting with chronic conditions was a challenge to frontline healthcare professionals in A&E. Nurse 3 describes concerns generated from feeling that they cannot perform their professional duties and responsibilities and properly look after the people in their care. In what follows, the effects of these conditions on healthcare professions, their identity and the sense of purpose of A&E staff will be discussed in further detail.

## Effects on healthcare professions: What is measured

Most participants discussed their perception that their profession had been negatively affected as a consequence of the austere environment described above. They talked about how meeting countable and measurable targets, such as the four-hour waiting time target has become the primary objective and that this pursuit was in tension with considerations about quality and immeasurable yet vital aspects of their work such as care and empathy:

“So it’s (the four-hour target) not an indicator of quality at all. […] it’s a bit of waffle isn’t it? And you could say, right ok, you are good quality because you get hardly any complaints, or incidents, or most patients have a safe journey through the A&E department. All those things are really important quality indicators. **So the performance target is just a measure of time. It is not a measure of anything else.** And they use it because it is easy to measure. **But you could argue that if people spend longer in the department, then they get more treatment, we may have more time to spend with them. They may get a better deal. So a four-hour target itself is not a quality measure.** (Doctor, 9) [Emphasis added]

Participants expressed discomfort and disquiet about having to make decisions about patient care based on improving flow through A&E rather than on patient needs. Doctor 5 recounted a recent scenario:

I know this patient needs an MR of their neck, I know it’s going to be very difficult to get it done tonight, it’s a low risk I am going to put in a special collar, send them home, bring them back tomorrow. **That’s something we don’t do in A&E**, we don’t bring people back… **when you do something out of the norm because you are trying to improve flow, it does feel uncomfortable at times… it feels uncomfortable to make decisions that are weighted on flow rather than clinical care.** (Doctor, 5) [Emphasis added]

There was a consensus among those interviewed that targets did not necessarily reflect overall quality of care, and, at times, obscured patient needs. As one nurse mentioned, the focus on quick turnaround times and rapid referrals made him *“feel a bit like working on a conveyor belt*” (Nurse, 15), while another participant described it as *“turning a profession into a mere job”* (Doctor, 5) as a ways of explaining the depersonalising effects of solely focusing on measurable targets rather than the quality of care provided.

Some participants mentioned that the four-hour target has helped A&E Departments negotiate for greater resources because it could be used to pressurise hospital management to pay attention to their requests. Furthermore, the introduction of some protocols were seen as positively helping improve the care of patients, e.g. the recently introduced protocol for detecting sepsis. However, the imposition of further standards and protocols was generally taken as signal of greater levels of micromanagement and task-oriented healthcare which alongside the aforementioned targets could detract from developing rapport and providing holistic care for patients. Participants perceived this to be contrary to the professional character of medical and nursing care, as it often forced them to “nurse the target” (Nurse, 6) rather than the patient.

The final sections demonstrate the effects of austerity on healthcare professionals themselves. They show how the limited availability of resources, healthcare funding cuts and the operationalisation of medical and nursing profession, impact on individuals working under these conditions.

## Effects on healthcare professionals: What or who is valued

A&E staff discussed that their efforts not to let down patients and not to let standards of care drop are uncounted and unappreciated by hospital management and by the political system in general. For instance, many of the nursing staff perceived the unavailability of funding for their continuous professional education and the slashing of their enhanced rate as an indication that the system does not value or appreciate their professions. There was evidence that the lack of funding is perceived as an affront by the A&E professionals in our study, as Nurse 3 said:

It feels very negative, like a very negative environment. It feels like we are not valued as a profession and that’s whether or not you are clinical facing or not. And **it seems to have gone worse since 2011.** Just the constant fighting for […] supplies, for funding for doing courses that you need for your job, **it’s just diminishing**. (Nurse, 3) [Emphasis added]

Many participants discussed pay as an indication of the extent to which they are valued by the healthcare system but many participants went to lengths to explain that their frustration was not connected with the *amount* of money they were receiving, but rather with the *discordance* between the type and amount of work and personal sacrifices they are asked to perform on a daily basis in relation to the pay they receive for it, as Doctor 8 reflects.

Every time that we go into a week knowing we are 5–6 doctors short because they would normally be filled by locum agencies and we can’t fill them because the Trust won’t pay money, or there isn’t the budget for supply doctors and we can’t recruit. **It’s just about feeling that actually our time, our own personal life is valuable, time with our families is valuable and we cannot expect to do over our contract every single working week, and increasingly so**. […]So I’ll happily go in and help when it is required, but for me personally and quite a few of my colleagues, **we don’t send a bill for overtime**. What we want is adequate rest […]. I think that’s really important to get across. **But yes we feel undervalued, but it’s not a monetary thing, it’s a recognition of our skill mix and a recognition of the stress which is upon us and the recognition of us as being human and having lives, which exist outside of the health service, which I think have long been forgotten by many people**.” (Doctor, 8) [Emphasis added]

The same sentiment was expressed by a number of interviewees, including the doctor speaking below, who stated that they would be happy with their salary as long as the physical and emotional labour performed is acknowledged and sufficient time is allowed for them to recover from the stress of being healthcare professionals.

“**if you are going to give someone all this stress to take home, then they are going to need, maybe not necessarily a high salary, but certainly time off to remunerate it, yes.** To be able to come down, off what was going on and the stress they are getting. I am not necessarily saying, get a higher salary, **I am saying if there was more money, then there would be more doctors and if there were more doctors, then that would mean if you had a really stressful night shift, then you wouldn’t have to be in work the next day and deal with something else you know**, or the next week or whatever. You know, you’ve got time to be able to do things to get your stress out.” (Doctor 16) [Emphasis added]

The pressure on healthcare professionals to work longer hours made them feel that their needs as employees were ignored. This had led to increased feelings of stress among the staff and burnout. Burnout denotes the feeling of mental, emotional and physical exhaustion due to prolonged involvement in situation that is emotionally demanding, leading to detachment, cynicism and a sense of ineffectiveness [57]. People working in caring professions like nursing and medicine, both physically and emotionally arduous occupations, are more prone to burnout; and this particularly true of healthcare professionals working in A&E. However, those interviewed felt that in recent years, feelings of burnout have increased:

**It was never a relaxed, easy going job but the intensity never used to be this high.** And you can see it just drains you emotionally, it drains you physically. **You go home broken a lot of the time**. And that’s really sad that a job … **I love my job I always loved my job, but it’s sad where it gets to the point where you think I cannot do this job anymore because I’m burnt out.** (Nurse, 1) [Emphasis added]

The toll of austerity at the level of the individual becomes clear in this quote. Not only did budgetary cuts affect the care given to individual patients but also the sense of professionalism experienced by individual members of A&E staff. Frustration, irritability and short temper are all common symptoms of burnout [56], signs of which are indicated in the above quote. However, such expressions and characteristics are seen as inconsistent with the role of a caring professional. Doctors and nurses trying to maintain professionalism during these adverse times, felt that this was at the expense of their personal, familial and collegial relationships, as Doctor 8 explains:

…part of being a doctor or nurse in an emergency department is to have that face that says, *“Hello I’m approachable*, *I’m here to help”*; which we all wear very well. If we couldn’t wear that face every day, the emergency department is not the right environment for us to be working in. […] **People become a little bit more blunt and even snappy with each other, not with patients, because again, you know, I think that none of us would want to be that way with patients**, but with our own friends and colleagues we can let our guard down a little bit, we can take away the professional face. And I think that is apparent, I see it with my nursing staff, I see it in my registrars and I see it increasingly within the consultant body that I work in. (Doctor, 8) [Emphasis added]

The quote below also talks about how the situation in A&E effects staff at all levels. Nurse 13 describes how knock-on effects of austerity policies elsewhere in the healthcare system are leading staff at the brink of physical and mental collapse:

“Yes definitely and **I don’t think that’s just necessarily at our level, that’s at every level, because it’s so full on**. **Because everything is now coming to us**, or it feels like it is, **because we can’t do the through put, because we have got people sat in our beds, burn out is huge.** And we have to be really careful of that and because people are doing the overtime and the extra, their burn out is high. […]**Emotion, anger, real frustration, absolute fatigue, throwing your arms up, can’t do any more. Yes, I think there is only so far you can push […]**.” (Nurse, 13) [Emphasis added]

When A&E professionals operate within a system that does not support their work, and hinders their ability to operate in ways that they believe to be compatible with their professional role, they experience a sense of disengagement and detachment. One doctor explains that after the realisation that their experience in A&E were part of larger, systemic problem that individual healthcare professionals cannot address, the doctor stopped trying to fix them to protect themselves from further burnout:

“**The harder decisions are what you do when the system is crashed around you.** So often **I will be down there, and maybe its unsafe, you know, there may be people dying, literally dying in the corridor and I used to worry about that too, but I don’t now** because what I do is email the duty manager and say, “you need to know it’s dangerous, you need to sort it out”” (Doctor, 7) [Emphasis added]+

Feelings of detachment and of powerlessness in the face of the conditions created by austerity policies were common among those interviewed. The longer one is confronted with situations where their professional integrity and ethos is compromised regularly, the more likely they are to suffer from moral and psychological effects such as detachments, low morale and burnout [57].

## Discussion

Working in A&E is by nature a stressful and emotionally demanding profession. Yet, from their experience and perspective, A&E staff in the UK are facing unprecedented challenges since the introduction of austerity policies. For many years their work has been defined by long hours, targets and the demands accompanied by being the front face of the NHS. Increased feelings of burnout and of being devalued and demoralised were reported as a part of their everyday experience. Furthermore, the numbers of socially vulnerable, elderly and mentally ill patients has increased in A&E. These changes in the demographic and pathological profile of patients have left staff feeling “impotent” as they are unable to provide appropriate and necessary care to these patients. As one participant mentioned, A&E staff feel that they *“… are in Catch 22*! *There is nowhere else for them (the patients) to go*.*”* In other words, staff feel that without the resources, power or authority to instigate substantive solutions to the issues they are facing, trying to care for patients in the current situation leaves them in an impossible situation. When A&E professionals are asked to operate in ways that they believe to be incompatible with their professional character, training and the ethos they consider appropriate to their role, they experience a sense of ‘erosion in values, dignity, spirit and will–an erosion of the human soul’ [57].

Our study found that although, from the position of those interviewed, austerity measures were not directly imposed on A&E, nevertheless the effects of austerity imposed on other areas of health and social care severely impacted on the A&E at departmental, professional, and individual levels. Our data confirms other studies that suggested that the continuous increase in admission is due to more chronic patients presenting at A&E as well as patients with more co-morbidities, but also because patients do not have access to appropriate care due to underfunding in areas such as mental health and social care [58]. A&E departments had to absorb the shortfall from other services and treat patients that in previous years would not have normally presented at the emergency departments. We found that the government’s evidence against claims of austerity in the NHS, contrasted the lived experience of the staff working in A&E departments. By choosing to measure the absolute amount of money given to the NHS, or the number of patients that ‘inappropriately’ presented in A&E, successive governments since 2010 have been able to mount a consistent defence against complaints about austerity in healthcare. However, we have found that a qualitative exploration of this issue has revealed a more nuanced picture of what counts as important for those on the frontline.

In our data, austerity policies and their effects have had also an impact on the healthcare professions. The increasing number of admissions in A&E was linked with a renewed attention and focus on targets. Although, targets in some cases can help improve patient care, they are not measures of care quality [59, 60]. A focus on measuring quantity (of patients) and time (e.g. time to admission, treatment or discharge) can obscure the quality of care provided, particularly to the types of patients with chronic issues that would not normally present in A&E, such as the elderly and the mental health patients. For example, the fact that an elderly patient is discharged from A&E within four hours of admission, does not necessarily indicate that this patient received the care she needed, which might include access to community care services.

It could be argued that the observed operationalisation of the healthcare profession, and the drive to meet targets (rather than meet patients) could help nurses and doctors perform better and more efficiently. By breaking down the personal relationship with patients, doctors and nurses could become less emotionally involved and hence provide better care to their patients. However, these changes have been interpreted by A&E staff as being contrary to what they perceive to be their professional role and professional ethos. This confirms earlier studies that looked at whether operationalisation and detachment could help improve performance [61], and aligns ideas of professionalism and professional ethos [62, 63].

Despite the relative small number of interviewees in this study, attention was paid to ensure a range of different healthcare professional roles and also of different locations across the country. This study confirms findings from larger reports and quantitative research, and also echoes similar findings regarding austerity and healthcare in other countries [44, 49]. More work would be necessary however, to fully capture and understand the changes austerity has instigated in healthcare at a systemic and professional level.

## Limitations of the study

We acknowledge the limited size of our study, which makes the extrapolation of more general arguments regarding the effects of austerity on A&E staff more difficult or even questionable. Unfortunately, practical reasons prevented us from including more sites and conducting more interviews, as originally planned. As explained above, it took more than a year to go through the ethics approval process and receive final permissions from the R&D officers of individual sites to contact their staff and arrange interviews. By November 2017, we had to make the decision to stop trying to recruit. However, a preliminary analysis of our collected data showed that we have reached data saturation, which aided that decision. Although we accept that further interviews could potentially have provided more interesting data, we were confident about the validity of the themes that emerged from our analysis as they corresponded and confirmed other quantitative studies and media reports regarding austerity and healthcare in the UK.

## Conclusion

Austerity policies have affected the socio-economic landscape of healthcare provision in the UK at the systemic, professional level and also at the personal level of healthcare professionals; and nowhere was this change more noticeable than the waiting rooms of A&E departments around the country. This paper has shown that despite claims that problems in A&E should not be attributed to austerity cuts, an emphasis on what is measured, counted or valued reveals a different narrative among healthcare professionals working in A&E departments in England. Even though our participants argued that the A&E budget was not reduced, austerity policies elsewhere in the welfare system were having a significant effect on the ability of these departments to deliver empathetic and high-quality care to patients. An increase demand for care from the population, and particularly from mentally ill patients and the elderly, meant that departments changed from Accident and Emergency departments to departments of “Anything and Everything”. Therefore, only counting the money going into A&E does not reveal the whole picture of what is happening on the ground. Also, the increased pressure on the staff further the target-orientation nature of the healthcare professions. Yet, as our participants noted, targets and particularly the four-hour target is suited for measuring quantity and time rather than quality of care. This way the quality of care patients are receiving can be concealed. This can be even more problematic when considering patient groups that would not normally present in A&E, such as the elderly and the mentally ill. At a personal level, healthcare professionals felt that the way they are perceived by the hospital management and also the political system has changed, expressing feelings of being devalued. The pressure to perform in an understaffed and under-resourced environment has led to increasing feelings of burnout and low morale.

This study comes to add an important and missing perspective from the ground to the growing literature regarding the effects of austerity in healthcare in the UK. It supports, with qualitative methods and theoretical analysis, the findings of the existing economic and quantitative studies and adds depth to the results of large scale studies. In this way in helps to better describe and understand the full effect of austerity in healthcare.
